# Vaccination Status in Pregnancy: A Cross‐Sectional Study of Two Greek Maternity Hospitals

**DOI:** 10.1155/jp/8993559

**Published:** 2026-10-06

**Authors:** Alexandros Psarris, Dimitris Baroutis, Rafail Mantzioros, Agni Pantou, Ioakeim Sapantzoglou, Michael Sindos, Marianna Theodora, Panagiotis Antsaklis, Pampanos Andreas, Aikaterini Gentsoglou, Afroditi Gorpa, Panayiotis D. Ziakas, George Daskalakis, Aristotelis Tsiakalos

**Affiliations:** ^1^ 1st Department of Obstetrics & Gynecology Alexandra Hospital National and Kapodistrian University of Athens Athens Greece uoa.gr; ^2^ Department of Genetics Alexandra Hospital Athens Greece hosp-alexandra.gr; ^3^ Leto General Obstetrics & Gynecological Clinic Athens Greece; ^4^ Brown University Providence Rhode Island USA brown.edu

**Keywords:** COVID-19, immunization, influenza, pregnancy, RSV, Tdap, vaccinations

## Abstract

**Purpose:**

This study is aimed at evaluating postpartum women′s vaccination status in two major maternity hospitals in Athens, Greece, and identifying demographic, socioeconomic, and behavioral factors influencing vaccine acceptance.

**Methods:**

A cross‐sectional study was conducted at Leto General Hospital and Alexandra Hospital. Postpartum women completed a questionnaire covering demographics, obstetric history, vaccination status (COVID‐19, tetanus–diphtheria–acellular pertussis [Tdap], influenza, and respiratory syncytial virus [RSV]), and reasons for vaccine acceptance or refusal. Data were analyzed to assess the potential factors associated with vaccination uptake.

**Results:**

A total of 725 postpartum women participated (Site 1: 531, Site 2: 194; median age: 34 years). Overall, 51.2% received at least one vaccine during pregnancy, with the highest uptake observed for Tdap (42.5%), followed by influenza (19.6%) and COVID‐19 vaccination (3.6%). RSV vaccination was negligible (< 1%). Influenza vaccination was significantly higher in Site 1 (22.2%) compared with Site 2 (12.4%, *p* < 0.001), whereas uptake rates for the other vaccines did not differ significantly between sites. Key barriers to vaccination included lack of physician advice (Site 1: 29.0%, Site 2: 18.6%), personal choice (Site 1: 12.4%, Site 2: 14.9%), and safety concerns, which were more prevalent in Site 2. Higher educational attainment and urban residence remained independently associated with vaccine uptake, after adjustment for potential confounding factors.

**Conclusion:**

Maternal immunization in Greece still remains a great challenge, since only 14.62% of the studied sample received multiple vaccines. Enhancing immunization strategies necessitates targeted interventions that address provider–patient communication, systemic barriers, and perceptions of vaccine safety in order to improve adherence to recommended vaccination schedules.

**What Does This Study Add to the Clinical Work?**

Despite the implementation of international recommendations in the national guidelines, the achievement of optimal vaccination rates is still under pursuit. Our study revealed significant factors potentially causing such a suboptimal coverage rate, factors that need to be further evaluated and addressed by the public and health providers to develop an appropriate monitoring system and targeted interventions.

## 1. Introduction

Maternal immunization has emerged as a critical public health strategy that provides dual protection for mothers and their infants against vaccine‐preventable diseases. The evolution of immunization recommendations has followed our growing understanding of the maternal immune system and its interaction with the fetus, as well as the recognition of the consequences of infectious diseases in pregnancy, including vertical transmission [1–3]. This approach has gained particular significance in response to recent public health challenges, including the COVID‐19 pandemic and the ongoing concerns regarding respiratory syncytial virus (RSV) infections in infants.

Recent scientific advancements have led to the development and approval of several vaccines that are specifically recommended for administration during pregnancy. The tetanus–diphtheria–acellular pertussis (Tdap) vaccine has demonstrated remarkable efficacy in preventing pertussis in young infants [4, 5], and maternal antibody transfer through vaccination has proven particularly crucial for protecting infants during their most vulnerable period before they can receive their own immunizations [6–8]. Influenza vaccination during pregnancy represents another cornerstone of maternal immunization. The inactivated influenza vaccine can be safely administered during any trimester of pregnancy, with no increased risk of major structural birth defects [9–11], and the provided protection extends to the infant through transplacental antibody transfer, providing crucial protection during the first 6 months of life when direct infant vaccination is not yet possible [12, 13].

The COVID‐19 pandemic brought unprecedented attention to maternal vaccination. Multiple studies have now confirmed that mothers with COVID‐19 face increased risks of severe complications such as higher rates of preterm birth and elevated mortality rates, whereas vertical transmission rates have been documented in the range of 3.9%–11.9% [14, 15]. Evidence has demonstrated that maternal vaccination effectively reduces rates of morbidity and hospitalization among infants aged less than 6 months [16, 17] and the safety and effectiveness of mRNA vaccines in pregnancy for both Delta and Omicron SARS‐CoV‐2 variants have been thoroughly investigated and confirmed [18–20]. Recently, the development of maternal RSV vaccination has marked a significant advancement in protecting infants from severe medically attended respiratory disease [21, 22], and two prevention strategies have emerged: maternal immunization and the administration of nirsevimab, a monoclonal antibody, to the newborn, both demonstrating exceptional efficacy in reducing infantile severe RSV‐associated LRTI [23].

Despite these scientific advancements and the clear recommendations issued both from scientific societies and health organizations, reaching an optimal vaccination coverage during pregnancy continues to be a global challenge. A number of factors modulate successful adoption and access to vaccines during pregnancy, including local and regional recommendations, financial constraints, vaccine availability, manufacturing capacity, and vaccine hesitancy among both providers and patients [24, 25]. Extensive data suggest that the achievement of the greatest maternal immunization possible relies on a high proportion of the susceptible population being immunized, a condition that depends on containing costs, optimizing vaccine manufacturing and supply chains, and maintaining a well‐organized health system offering comprehensive, ideally free‐of‐charge immunization programs [26].

The Greek healthcare system faces unique challenges in achieving such optimal coverage, even though protocols and recommendations, aligned with current evidence [27–40], are in place. However, actual vaccination rates seem to continue to be significantly low in the Greek population, and factors influencing vaccine acceptance still remain inscrutable. The main objective of our study was to evaluate postpartum women′s vaccination status in two major maternity hospitals in Athens, Greece, and identify demographic, socioeconomic, and behavioral factors influencing vaccine acceptance.

## 2. Materials and Methods

### 2.1. Study Design and Setting

A cross‐sectional study was conducted at Leto Hospital (Site 1) and Alexandra Hospital (Site 2) in Athens, Greece, between May 2024 and October 2024. The two maternity hospitals serve the most populated urban area in Greece, consisting of the Athens metropolitan area and Attica region, a home for 36.38% of the Greek population and 38.67% of females of reproductive age [41]. These institutions were selected as they represent two of the largest maternal healthcare centers in Athens, serving diverse patient populations. Leto Hospital, located in a northern suburb of Attica, serves a maternal population with higher income and socioeconomic status. Alexandra Hospital, situated in the center of Athens, serves a more diverse population, offering a broader range of general healthcare services. Patients mostly live in the hospital′s surrounding neighborhoods, with the average travel distance being approximately 5–15 km from their homes. Therefore, access to healthcare services at Alexandra Hospital may contribute to a broader patient demographic. The study protocol received approval from the Research Ethics Committee at both participating hospitals (Alexandra Hospital: 277/2024, Leto Maternity Hospital: 188/2024). Data anonymization was secured throughout the study period, and no compensation was provided for participation in the study.

### 2.2. Study Sample Size

The study sample consisted of postpartum women who delivered at either participating hospital during the defined study period. No formal sample size calculation was performed; instead, we adopted a convenience sampling approach that included all eligible postpartum women who delivered during the 6‐month study period and met the inclusion criteria. Following established methodologies from recent European vaccination studies [42, 43], eligible participants included women who delivered a live infant at 37 weeks of gestation or later, were able to read and write in Greek, were aged 18 years or older, and provided informed consent. Exclusion criteria included women under 18 years of age, women with insufficient proficiency in the Greek language, individuals with cognitive impairments affecting their ability to provide consent, and cases of delivery prior to 37 weeks of gestation. None of the women that were approached to be included in the study refused to participate. This sampling approach is aimed at capturing a comprehensive representation of the maternal population at both institutions during the study period.

### 2.3. Data Collection Instrument

A structured questionnaire was developed through a comprehensive literature review and expert consultation. The draft questionnaire was reviewed by members of the study team with expertise in obstetrics and gynecology and infectious diseases for clarity, relevance, and comprehensibility, and their feedback was incorporated into the final version prior to administration. As the instrument was designed to collect demographic, obstetric, and vaccination‐related information, no formal validation or separate pilot‐testing phase was undertaken. The final questionnaire encompassed demographic information, obstetric history, vaccination status during pregnancy, knowledge and attitudes toward vaccination, and reasons for vaccine acceptance or refusal. Demographic data included age, ethnicity, residence location, educational level, and employment status. Obstetric history covered current pregnancy characteristics. Vaccination status assessment included detailed information about COVID‐19, Tdap, influenza, and RSV vaccination, along with timing and other vaccines received during pregnancy.

### 2.4. Data Collection Procedure

Trained research obstetrics and gynecology residents and midwives approached eligible participants during their postpartum hospital stay, typically between 24 and 48 h after delivery. Participants received information about the study purposes, assured data confidentiality, and provided written informed consent before completing the self‐administered questionnaire. The average completion time was 10 min, with assistance available for clarification, if needed.

Quality control measures included regular training sessions for research assistants, standardized collection procedures, daily review of completed questionnaires, double data entry by two independent doctors, and weekly team meetings to address concerns. All data were stored securely in password‐protected databases, accessible only to authorized research team members.

### 2.5. Statistical Analysis

Continuous outcomes were summarized as median (interquartile range [IQR]). Count outcomes were reported as frequencies (%) with 95% confidence intervals (95% CIs) and compared across groups using the chi‐square test. Logistic regression analysis was performed to identify factors associated with receipt of at least one vaccine during pregnancy. Variables associated with vaccine uptake in univariate analysis were entered into a multiple logistic regression model adjusting for study site, maternal age, residence, and educational level. Results were reported as odds ratios (ORs) and adjusted odds ratios (aORs) with corresponding 95% CIs. Level of significance was set to *α* = 0.05.

## 3. Results

A total of 725 postpartum women participated in this study across two sites (531 in Site 1 and 194 in Site 2). Table 1 provides a comprehensive overview of the demographic characteristics and vaccination rates observed in our study sample.

**Table 1:** Summary of key demographic characteristics and vaccination rates by study site.

Metric	Overall	Site 1	Site 2	*p*value
Sample size	725	531	194	—
Median age, years (IQR)	34.0 (30.0–37.0)	34.0 (30.5–38.0)	32.0 (28.0–36.0)	< 0.001
Urban residence	546/718 (76.0%)	412/525 (78.5%)	134/193 (69.4%)	0.016
Education level				< 0.001
University degree	304/716 (42.5%)	227/526 (43.2%)	77/190 (40.5%)	
PhD/master′s	177/716 (24.7%)	146/526 (27.8%)	31/190 (16.3%)	
Secondary education	215/716 (30.0%)	146/526 (27.8%)	69/190 (36.3%)	
Elementary only	20/716 (2.8%)	7/526 (1.3%)	13/190 (6.8%)	
Vaccination rates				
Overall (≥ 1 vaccine)	371/725 (51.2%, 95% CI: 47.5–54.8)	270/531 (50.8%, 95% CI: 46.6–55.1)	101/194 (52.1%, 95% CI: 45.0–59.0)	0.84
Tdap	308/725 (42.5%, 95% CI: 38.9–46.1)	216/531 (40.7%, 95% CI: 36.6–45.0)	92/194 (47.4%, 95% CI: 40.5–54.4)	0.123
Influenza	142/725 (19.6%, 95% CI: 16.9–22.6)	118/531 (22.2%, 95% CI: 18.9–25.9)	24/194 (12.4%, 95% CI: 8.5–17.6)	< 0.001
COVID‐19	26/725 (3.6%, 95% CI: 2.5–5.2)	16/531 (3.0%, 95% CI: 1.9–4.9)	10/194 (5.2%, 95% CI: 2.8–9.2)	0.25
Multiple vaccines	106/725 (14.6%, 95% CI: 12.2–17.4)	80/531 (15.1%, 95% CI: 12.3–18.3)	26/194 (13.4%, 95% CI: 9.3–18.9)	0.58
Tdap + influenza	83 (78.3%, 95% CI: 69.5–85.1)	68 (85.0%, 95% CI: 75.8–91.1)	15 (57.7%, 95% CI: 38.9–74.5)	
Influenza + COVID‐19	5 (4.7%, 95% CI: 2.0–10.6)	3 (3.8%, 95% CI: 1.3–10.5)	2 (7.7%, 95% CI: 2.1–24.1)	
Tdap + COVID‐19	9 (8.5%, 95% CI: 4.5–15.4)	3 (3.8%, 95% CI: 1.3–10.5)	6 (23.1%, 95% CI: 11.0–42.1)	
Tdap + influenza + COVID‐19	6 (5.7%, 95% CI: 2.6–11.8)	4 (5.0%, 95% CI: 2.0–12.1)	2 (7.7%, 95% CI: 2.1–24.1)	
Tdap + influenza + RSV	1 (0.9%, 95% CI: 0.2–5.1)	1 (1.3%, 95% CI: 0.2–6.8)	0	
Tdap + RSV	1 (0.9%, 95% CI: 0.2–5.1)	0	1 (3.8%, 95% CI: 0.7–18.9)	
Tdap + influenza + COVID‐19 + RSV	1 (0.9%, 95% CI: 0.2–5.1)	1 (1.3%, 95% CI: 0.2–6.8)	0	

*Note:* Percentages were calculated using available‐case denominators where missing data existed. *p* values derived from *χ* ^2^ tests for categorical variables and the Mann–Whitney *U* test for age.

Abbreviations: CI, confidence interval; IQR, interquartile range; Tdap, tetanus–diphtheria–acellular pertussis vaccine.

The study sample had a median age of 34.0 years (IQR: 30.0–37.0; range: 18.0–50.0), with 76.0% residing in urban areas. The majority of participants (91.1%) self‐identified as being of Greek ethnic origin, followed by 5.0% of Albanian ethnicity, whereas the remaining participants represented various European, African, and Asian ancestries. Regarding educational attainment, 42.5% held a university degree, 24.7% had completed PhD/master′s programs, 30.0% had completed secondary education, and 2.8% had only elementary education.

The educational distribution of the study sample was broadly comparable to that of the female census population, with the exception of PhD/master′s degree holders, who were overrepresented in our sample.

As shown in Figure 1, overall vaccination coverage revealed that 51.17% of participants received at least one of the designated vaccines. The highest uptake was observed for Tdap vaccination (42.48%), followed by influenza (19.58%) and COVID‐19 (3.59%). RSV vaccination rates were negligible (< 1%) and therefore not included in the graphical representation.

**Figure 1:**
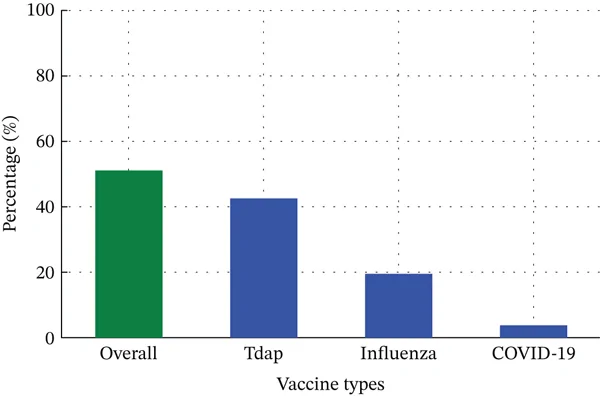
Overall vaccination rates by vaccine type.

When comparing vaccination rates between sites (Figure 2), the overall rates of receiving at least one vaccine were similar between Site 1 (50.85%, 95% CI: 46.60–55.10) and Site 2 (52.06%, 95% CI: 45.03–59.09, *p* = 0.84). Significant differences were observed in influenza vaccination rates, with Site 1 showing higher coverage (22.22%, 95% CI: 18.69–25.76) compared with Site 2 (12.37%, 95% CI: 7.74–17.00, *p* < 0.001). Other vaccination rates showed no statistically significant differences between sites: Tdap vaccination rates were 40.68% (95% CI: 36.50–44.86) at Site 1 and 47.42% (95% CI: 40.40–54.45) at Site 2 (*p* = 0.123), whereas COVID‐19 vaccination rates were 3.01% (95% CI: 1.56–4.47) at Site 1 and 5.15% (95% CI: 2.04–8.27) at Site 2 (*p* = 0.25).

**Figure 2:**
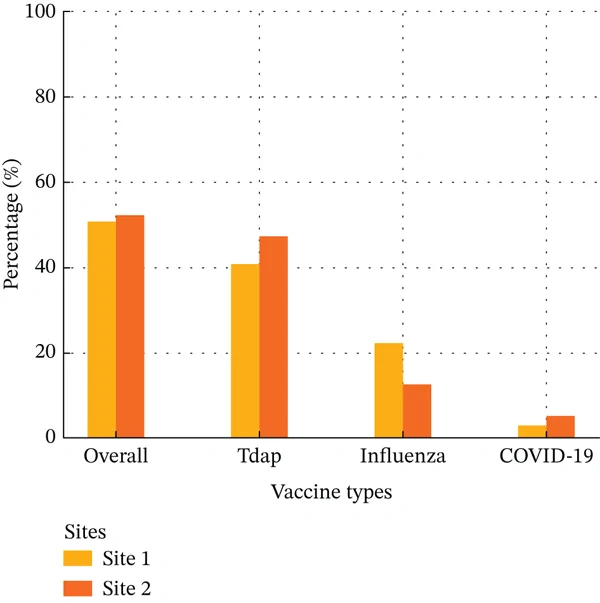
Comparison of vaccination rates between sites.

Analysis of multiple vaccine uptake revealed that 14.62% (106/725) of participants received two or more vaccines, including 0.96% (7/725) who received three vaccines and only one participant who received all four vaccines.

Regarding reasons for nonvaccination (Figure 3), the distribution differed significantly between sites (*p* < 0.001). The predominant reason at both sites was lack of clear medical advice (Site 1: 29.0%, Site 2: 18.6%) followed by personal choice (Site 1: 12.4%, Site 2: 14.9%). Notable differences between sites were observed in security concerns (Site 2: 8.2% vs. Site 1: 1.3%) and incomplete follow‐up (Site 2: 4.1% vs. Site 1: 0.6%). Conversely, nonresponse rates were higher in Site 1 compared with Site 2 (6.4% vs. 0.5%).

**Figure 3:**
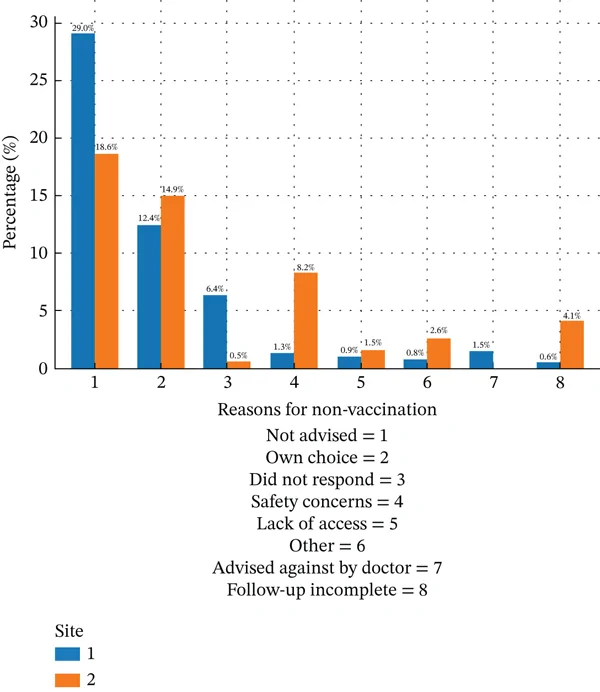
Reasons for nonvaccination by site.

In univariate analysis, older maternal age was associated with increased odds of receiving at least one vaccine during pregnancy (OR 1.04 per year increase, 95% CI: 1.02–1.07, *p* = 0.002). Women residing in urban areas were also more likely to be vaccinated compared with those in rural areas (OR 1.66, 95% CI: 1.17–2.36, *p* = 0.004). Educational attainment was associated with vaccine uptake, with women holding secondary education (OR 7.25, 95% CI: 1.64–32.01, *p* = 0.009), university degrees (OR 10.35, 95% CI: 2.36–45.39, *p* = 0.002), and PhD/master′s degrees (OR 14.51, 95% CI: 3.26–64.52, *p* < 0.001) having higher odds of vaccination compared with women with elementary education. Study site was not significantly associated with vaccine uptake (OR 1.04, 95% CI: 0.75–1.45, *p* = 0.807).

In the multiple logistic regression model adjusted for study site, maternal age, residence, and educational level, higher educational attainment remained the strongest predictor of vaccination. Compared with women with elementary education, those with secondary education (aOR 5.63, 95% CI: 1.24–25.62, *p* = 0.025), university education (aOR 7.73, 95% CI: 1.70–35.20, *p* = 0.008), and PhD/master′s degrees (aOR 10.66, 95% CI: 2.29–49.52, *p* = 0.003) had higher odds to receive at least one vaccine during pregnancy. Urban residence also remained independently associated with vaccine uptake (aOR 1.45, 95% CI: 1.01–2.09, *p* = 0.045), whereas maternal age showed a borderline association after adjustment (aOR 1.03, 95% CI: 1.00–1.06, *p* = 0.052). Study site remained nonsignificant in the adjusted model (aOR 1.33, 95% CI: 0.94–1.90, *p* = 0.110).

## 4. Discussion

### 4.1. Main Findings of Our Study

This cross‐sectional study addresses a critical gap in knowledge regarding maternal vaccination patterns in Greece, particularly considering the evolving landscape of maternal immunization. Our findings provide unique insights into factors influencing vaccine acceptance across different contexts and contribute to our growing understanding of maternal–fetal immunology and pregnancy‐associated infectious disease risks [44–46].

The overall vaccination rate of 51.17% demonstrates significant room for improvement in maternal immunization coverage. Recent scientific advances and evidence consistently support the crucial role of maternal vaccination in protecting both mother and infant [47, 48]. The variations in uptake between different vaccines and study sites provide valuable insights into both systemic and individual barriers to maternal immunization. Higher educational level remained the strongest independent predictor of vaccination, whereas urban residence was also positively associated with vaccine uptake. Maternal age showed a borderline association after adjustment, whereas study site remained nonsignificant. These findings suggest that socioeconomic and educational factors were the primary determinants of maternal vaccination uptake in this cohort. Among women receiving multiple vaccinations, adherence largely follows the established recommendations for Tdap and seasonal influenza vaccination, whereas uptake of COVID‐19 and RSV vaccines remained limited.

### 4.2. Comparison to Existing Literature

Vaccines such as Tdap, influenza, COVID‐19, and RSV are essential components of a comprehensive strategy to protect both maternal and neonatal health [1]. However, despite their proven safety and efficacy, challenges such as vaccine hesitancy, misinformation, and limited access continue to hinder the achievement of high vaccination rates among pregnant individuals [49, 50].

Our observed Tdap uptake of 42.5% falls considerably short of optimal coverage needed for effective protection against pertussis in newborns. The variation between sites (40.68% vs. 47.42%) might suggest that several institutional factors (hospital protocols, presence of booklets and posters, and trained or sensitized personnel) may influence vaccination rates, though not reaching statistical significance.

The notably lower influenza vaccination rate (19.58%) with significant disparity between sites (Site 1: 22.22% vs. Site 2: 12.37%, *p* < 0.001) raises concerns given that pregnant women face elevated risks of severe complications from influenza [51]. Studies have consistently shown that vaccination substantially reduces both maternal and neonatal morbidity with no increased risk of major structural birth defects [51, 52].

Furthermore, the COVID‐19 pandemic has underscored the critical importance of vaccination during pregnancy [45]. Health organizations, including the Centers for Disease Control and Prevention (CDC) and the World Health Organization (WHO), strongly recommend COVID‐19 vaccination for pregnant individuals, given the benefits of preventing severe disease and protecting both mother and child [47, 53]. The remarkably low COVID‐19 vaccination rate reported in our study (3.59%) warrants careful consideration, especially given the documented vertical transmission rates of 3.9%–11.9% and the increased risks of severe complications in pregnant women [54, 55]. Multiple studies have confirmed the safety and efficacy of the mRNA vaccine in pregnancy, making this low uptake particularly concerning [56].

In view of RSV, the negligible vaccination rate (< 1%) likely reflects its recent introduction in the maternal immunization guidelines and the fact that it is not still compensated by the Greek Ministry of Health. The absence of vaccinated mothers requires urgent attention given that approximately 30% of infants develop lower respiratory tract infections in their first year [35].

Analysis of reasons for nonvaccination provides crucial insights for improving coverage. The predominant barrier—lack of clear medical advice (29.0% in Site 1, 18.6% in Site 2)—suggests a critical need for improved healthcare provider communication, and the variation in safety concerns between sites (1.3% vs. 8.2%) reveals that educational interventions may need to be tailored to specific institutional contexts.

It is well established that successful maternal immunization programs require high population coverage and depend on multiple interconnected factors including cost management, supply chain optimization, healthcare system organization, and targeted strategies in order to address and deal with vaccine hesitancy [50, 57]. To further underline such hesitancy, the fact that in our cohort only 14.62% received multiple vaccines highlights the need for comprehensive approaches to maternal vaccination.

### 4.3. Strengths and Limitations

The main strength of our study is its multicentric design and the diverse population that was included in terms of socioeconomic and educational level. Our sample size, albeit relatively small, corresponds to more than 1% of all pregnant women during 2024, taking into account that the total number of births in Greece was 71,455.

However, several limitations should be considered when interpreting these results. Our data included a modest sample size of 725 pregnant women, and the cross‐sectional design of our study limits causal inference. Furthermore, lack of clear medical advice and consultation was demonstrated as one of the predominant reasons for nonvaccination. However, provider recommendations were not independently verified, and as such, the true impact and effect of medical guidance (or the lack of it) on the subsequent vaccination rates can only be hypothesized. Similar to the above, institutional factors may explain differences between study sites, but, given the fact that study site was not independently associated with vaccine uptake in the adjusted analysis, such a finding may only be hypothesized rather than definitely concluded. Furthermore, the two‐site urban setting excluded the rural population and the management that takes place in rural health facilities, a condition that limits the generalizability of our findings in the broader Greek population. Furthermore, our study used convenience sampling, and, as such, our study is subject to selection bias. In addition to the above, the vaccination status was self‐reported, introducing the risk of recall bias in our dataset.

### 4.4. Future Research and Directions

Our findings support the development of targeted interventions to optimize vaccination coverage during pregnancy. The research on vaccine hesitancy suggests focusing on healthcare provider education, culturally appropriate communication strategies, and systematic monitoring of vaccination coverage [58, 59].

Our collective effort is ongoing to recruit new sites and participants. Future research directions should include the following:

1. Longitudinal studies examining vaccination patterns over time
2. Investigation of healthcare provider perspectives
3. Evaluation of targeted interventions to improve vaccine uptake
4. Assessment of cost‐effectiveness for different intervention strategies
5. Development of standardized protocols across healthcare institutions

This work establishes a foundation for future investigations and interventions aimed at improving maternal immunization programs in Greece and similar healthcare contexts. The demonstrated variations in vaccination rates and barriers between sites highlight the need for both system‐level changes and targeted interventions to optimize coverage and reduce the burden of vaccine‐preventable diseases in these vulnerable populations.

## 5. Conclusion

This comprehensive assessment of maternal vaccination patterns in Greece reveals significant challenges with overall vaccination rates of 51.17% for at least one vaccine and notably low uptake for specific vaccines like COVID‐19 (3.59%) and RSV (< 1%). Higher educational level remained the strongest independent predictor of vaccination, whereas urban residence was also positively associated with vaccine uptake. Key barriers identified include insufficient medical advice highlighting, as such, the need for improved healthcare provider education and standardized vaccination protocols.

To enhance maternal vaccination programs in Greece, a coordinated approach involving healthcare institutions, providers, and public health authorities is essential. Future efforts should focus on developing culturally appropriate communication strategies, implementing robust monitoring systems, and conducting further research to evaluate targeted interventions. These evidence‐based improvements will be crucial for optimizing vaccine coverage and better protecting maternal and infant health across the country.

## Author Contributions

Protocol/project development: Dimitris Baroutis, Aristotelis Tsiakalos, and Alexandros Psarris; methodology: Dimitris Baroutis, Alexandros Psarris, Agni Pantou, Rafail Mantzioros, and Aristotelis Tsiakalos; data analysis: Dimitris Baroutis, Alexandros Psarris, Panagiotis Antsaklis, Michael Sindos, and Marianna Theodora; formal analysis: Dimitris Baroutis, Alexandros Psarris, Panagiotis Antsaklis, Ioakeim Sapantzoglou, and Panayiotis D. Ziakas; investigation: Dimitris Baroutis, Alexandros Psarris, and Aikaterini Gentsoglou; resources: Aikaterini Gentsoglou, George Daskalakis, and Aristotelis Tsiakalos; data curation: Rafail Mantzioros, Alexandros Psarris, Michael Sindos, and Panayiotis D. Ziakas; manuscript writing/editing: Dimitris Baroutis, Ioakeim Sapantzoglou, and Alexandros Psarris; writing—review and editing: Alexandros Psarris, Aristotelis Tsiakalos, and Agni Pantou; visualization: Marianna Theodora and Panagiotis Antsaklis; supervision: George Daskalakis and Aristotelis Tsiakalos; project administration: Dimitris Baroutis and Alexandros Psarris.

## Funding

No funding was received for this manuscript.

## Disclosure

The authors affirm that human research participants provided informed consent for publication. All authors have read and agreed to the published version of the manuscript.

## Ethics Statement

The study was conducted in accordance with the Declaration of Helsinki and was approved by the Institutional Review Boards of Leto and Alexandra General Hospitals of Athens, Greece.

## Consent

Informed consent was obtained from all subjects involved in the study.

## Conflicts of Interest

The authors declare no conflicts of interest.

## Supporting information


**Supporting Information** Additional supporting information can be found online in the Supporting Information section. Structured questionnaire.

## Data Availability

The data that support the findings of this study are available on request from the corresponding author. The data are not publicly available due to privacy or ethical restrictions.
