# Supplementary material for: Vaccination Status in Pregnancy: A Cross‐Sectional Study of Two Greek Maternity Hospitals
Source: J Pregnancy. 2026 Oct 6;2026:8993559. doi: 10.1155/jp/8993559 (PMC13639827; doi:10.1155/jp/8993559)
Supplement: Supplementary file 1 [file JP-2026-8993559-s001.docx]

Questionnaire

- Have you been vaccinated during your childhood according to the National Vaccination Schedule?

Yes	No	Don’t know/Don’t want to answer

- Are you planning to vaccinate your child according to the pediatrician’s instructions?

Yes	No	Don’t know/Don’t want to answer

- Would you get vaccinated during pregnancy if your doctor recommended it?

Yes	No	Don’t know/Don’t want to answer

- Did your doctor recommend any vaccinations during pregnancy?

Yes	No	Don’t know/Don’t want to answer

- Against which disease?

Flu	Pertussis	Other …………………………………..

- Were you vaccinated during pregnancy?

Yes	No	Don’t know/Don’t want to answer

- Which disease were you vaccinated against?

Flu	Pertussis	Other …………………………………..

- In case you were NOT vaccinated during pregnancy, which was the reason?

My doctor didn’t recommend any vaccins during pregnancy

I was afraid of possible side effects on myself

I was afraid of possible side effects on the fetus

Other …………………………………..

Full Name:

ID:

Medical History:

Age:

Gestational Age at Birth:

Obstetrician’s full name:
